# Supplementary material for: The interactome of intact mitochondria by cross-linking mass spectrometry provides evidence for coexisting respiratory supercomplexes
Source: Mol Cell Proteomics. 2017 Dec 8;17(2):216–32. doi: 10.1074/mcp.RA117.000470 (PMC5795388; doi:10.1074/mcp.RA117.000470)
Supplement: Supplemental Data [file supp_RA117.000470_133922_0_supp_23978_fzffwf.zip › spectra_annotation/mito_spectra_annotation/44-1-4-1-3-1.pdf]

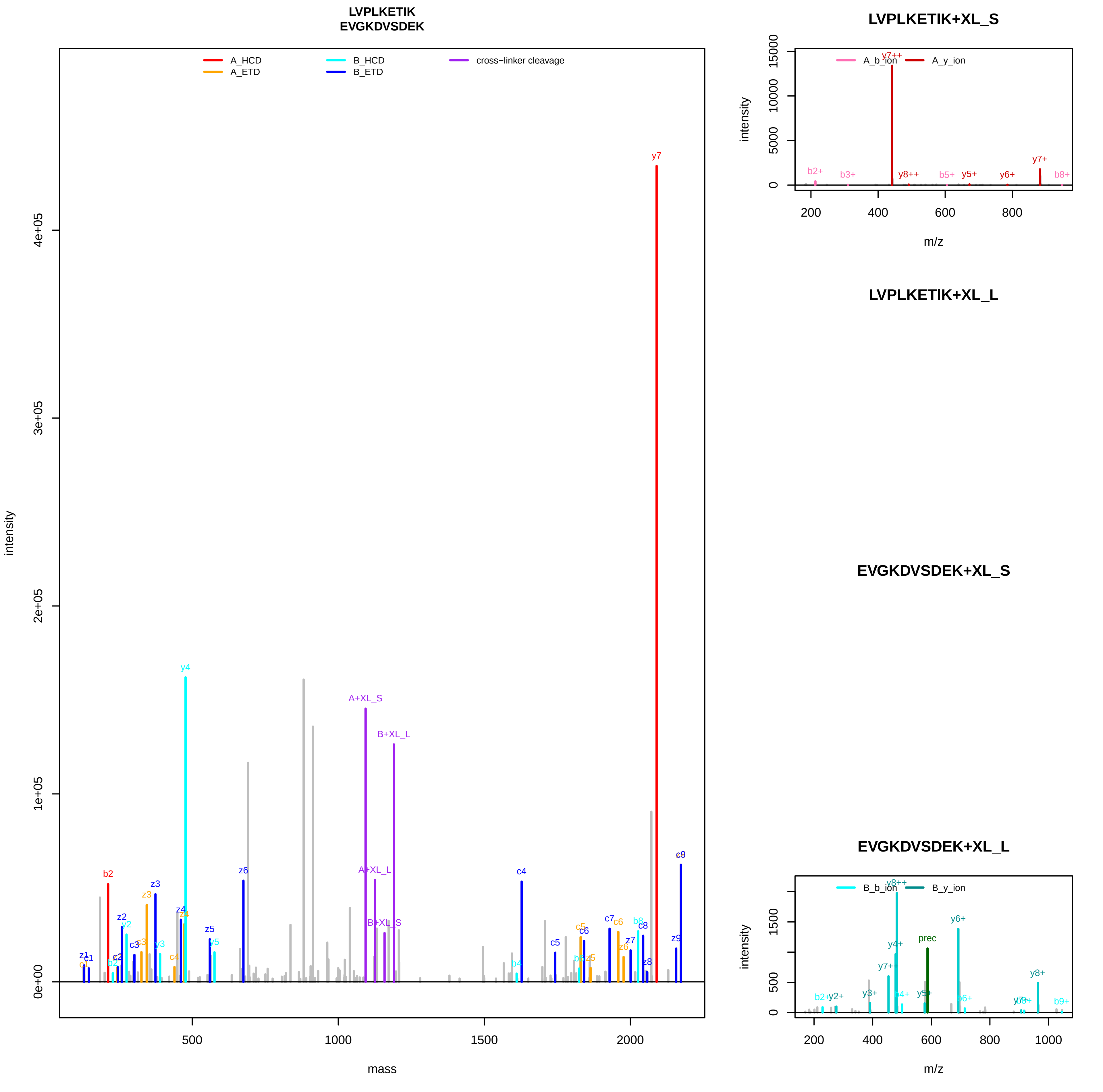
LVPLKETIK
EVGKDVSDEK
A_HCD
A_ETD
B_HCD
B_ETD
cross−linker cleavage
intensity
mass
0e+00
1e+05
2e+05
3e+05
4e+05
500
1000
1500
2000
y7
y4
A+XL_S
B+XL_L
A+XL_L
B+XL_S
b2
z1
c1
c2
z2
y2
c3
z3
y3
z4
z5
y5
z6
c4
b4
c5
c6
c7
z7
c8
b8
z8
z9
c9
LVPLKETIK+XL_S
A_b_ion
A_y_ion
y7++
b2+
b3+
y8++
b5+
y5+
y6+
y7+
b8+
m/z
0
5000
10000
15000
200
400
600
800
LVPLKETIK+XL_L
EVGKDVSDEK+XL_S
EVGKDVSDEK+XL_L
B_b_ion
B_y_ion
y8++
y6+
prec
y4+
y7++
y3+
y5+
b2+
y2+
y8+
b9+
0
500
1500
1000
